# Fused Filament Fabrication of Polyethylene/Graphene Composites for In-Space Manufacturing

**DOI:** 10.3390/ma17081888

**Published:** 2024-04-19

**Authors:** Susanna Laurenzi, Federica Zaccardi, Elisa Toto, Maria Gabriella Santonicola, Sabina Botti, Tanya Scalia

**Affiliations:** 1 Department of Astronautical Electrical and Energy Engineering, Sapienza University of Rome, Via Salaria 851-881, 00138 Rome, Italy; federica.zaccardi@gmail.com; 2 Department of Chemical Engineering Materials Environment, Sapienza University of Rome, Via del Castro Laurenziano 7, 00161 Rome, Italy; elisa.toto@uniroma1.it (E.T.); mariagabriella.santonicola@uniroma1.it (M.G.S.); 3 Photonics Micro- and Nano-Structures Laboratory, Division of Physical Technologies and Security, Nuclear Department, ENEA C.R. Frascati, Via E. Fermi 45, 00044 Frascati, Italy; sabina.botti@enea.it; 4 Italian Space Agency, Via del Politecnico s.n.c., 00133 Rome, Italy; tanya.scalia@asi.it

**Keywords:** 3D printing, composites, graphene, polyethylene, space environment

## Abstract

Graphene-based composite materials are highly sought after for space applications due to their ability to encompass various properties, such as electrical conductivity, thermal resistance, and radiation shielding. This versatility allows for the creation of multifunctional components that can serve various purposes in space. Three-dimensional (3D) printing of composite materials in space offers a versatile and efficient means of manufacturing components, tools, and structures that are tailored to the unique challenges and requirements of space missions. In this work, we aim to develop 3D-printed composites made of medium-density polyethylene (MDPE) matrix and exfoliated graphene nanoplatelets (xGnP) as filler, using fused filament fabrication (FFF). Our research focuses on the challenges associated with the FFF process for fabricating MDPE/xGnP materials, particularly by optimizing filament extrusion and assessing the resulting material properties and space environmental compatibility. Firstly, we optimize the extrusion process, and use the MDPE/xGnP filaments to fabricate 3D-printed samples after defining the FFF parameters. We employ differential scanning calorimetry (DSC) to assess the melting properties and crystallization degree of the extruded filaments and 3D-printed samples, providing insights into the relationship between these properties and the characteristics of the initial powders. Electrical and tensile tests are carried out to evaluate the material properties after successfully mitigating challenges, such as warping and inadequate adhesion, to build plates during the printing process. Finally, we subject the 3D-printed composites to outgassing tests under exposure to the AM0 solar spectrum to evaluate their space environmental suitability. The results of this work demonstrate the capability of the FFF-based process to efficiently manufacture components made of MDPE/xGnP composites, providing optimized parameters for their potential in-space fabrication.

## 1. Introduction

Additive manufacturing (AM) of carbon-based composites via 3D printing is a sought-after technology for long-term manned missions. Three-dimensional printing allows for the on-site fabrication of components and tools, which is crucial for adapting to unforeseen challenges or repairs during space missions [1,2,3]. Composite materials can be tailored to specific mission requirements, offering flexibility in design and manufacturing [4]. Using 3D printing with composite materials aligns with the principles of sustainable space exploration by minimizing waste and reducing the environmental impact of space missions [1,5].

The 3D printing technology acquired attention in the aerospace field for the ability to customize geometry with high precision [6,7]. It has even been employed for producing components aboard the International Space Station (ISS) [8,9]. More recently, in view of long-term manned missions to the Moon and Mars, 3D printing presents an intriguing potential for processing local materials to construct habitats on-site [10,11]. One crucial aspect of this endeavor is In Situ Resource Utilization (ISRU), which involves harnessing the potential of hardware and materials brought along for the mission but no longer needed upon arrival, such as lander parts, electronic components, or packaging materials. These scavenged materials or hardware components can be processed or combined to create necessary equipment at the mission destination [1]. This could encompass maintenance tools, utensils, habitat components, or research instruments. The development of viable concepts for hardware recycling is thus essential for ensuring the sustainability of long-term human exploration missions.

Among the various additive 3D printing techniques, fused filament fabrication (FFF) stands out as the most promising technology for space applications [1,3]. This distinction arises from the fact that FFF does not necessitate the use of solvents or binding agents to create components [12] and can be successfully adopted in a zero-gravity environment [3,13]. Moreover, in contrast to other 3D printing methods, FFF-printed parts can be readily recycled, aligning with circular and sustainable practices. This recycling capability reduces the risks and logistical challenges associated with extended space missions. In fact, it is not coincidental that FFF was the chosen additive manufacturing process for investigation in space, specifically aboard the International Space Station (ISS), during the NASA Zero G Technology Demonstration Mission [13]. This mission involved the production and analysis of specimens crafted from acrylonitrile butadiene styrene (ABS), showcasing the suitability of FFF for in-space manufacturing and its potential to contribute to the sustainability and efficiency of space exploration endeavors. In the fused filament fabrication (FFF) process, the filament is heated above its melting temperature (T_m_) and then extruded layer-by-layer to construct a three-dimensional object based on the CAD model. Despite the seemingly straightforward nature of this manufacturing process, FFF demands precise optimization of process parameters to prevent various types of defects in the 3D-printed parts, such as gaps [14], overlaps, or offset issues [15]. Moreover, Kechagias et al. [16] highlighted the influence of FFF parameters on the porosity of printed parts, examining crucial results varying factors such as the infill type, part orientation, and the infill rate.

Specific mechanical requirements for 3D printing in space include maintaining precise control over the process to ensure accurate fabrication of components. They should be fabricated to withstand the harsh conditions of space and minimize the risk of failure or malfunction. The 3D printing process should be resource-efficient, requiring minimal energy and raw materials while maximizing production output. This involves optimizing printing parameters, recycling waste materials, or implementing closed-loop systems for material use. Moreover, the 3D printing technology in space occurs in microgravity conditions. This may require modifications to the printing process, such as implementing stabilizing mechanisms to ensure layer adhesion and dimensional accuracy. Polyethylene (PE) and its composites are the most appropriate materials for creating effective radiation barriers across a range of industries, including power generation [17], medicine [18], and aerospace [19]. Moreover, employing 3D printing to process PE waste can present an environmentally friendly solution for sustainable component production, while also delivering cost savings [20]. However, PE is a semi-crystalline material prone to significant shrinkage and warping during the solidification phase [21]. Additionally, the processability of PE via the FFF technique is limited by its compatibility issues with most build plate materials, often resulting in detachment of the printed part. Studies on the FFF process of PE have been carried out by different authors. Blends of high-density polyethylene (HDPE) or ultra-high molecular weight polyethylene (UHMWPE) combined with other thermoplastic polymers, such as acrylonitrile butadiene styrene (ABS) and polycarbonate-urethane (PCU), have been investigated while trying to solve the process issues. For example, Borges et al. studied the potential of the FFF 3D printing technology to fabricate an artificial knee meniscus made of PCU and UHMWPE [22], and they found that the inherent surface roughness of the 3D-printed surfaces is still a challenging issue to overcome, and improvements in the FFF technology are necessary. In this regard, a recent study [23] focused on the effects of 3D printing parameters on surface roughness, considering crucial factors such as the layer height, flow rate, and nozzle temperature. The results of this study can be partly applied to different materials to improve the quality of printed parts. Harris et al. proposed a ABS/HDPE blend to improve the thermal stability of ABS in the FFF process [24]. However, the presence of HDPE compromised the quality of the filaments and the mechanical properties of the printed parts [25]. Recently, Schirmeister et al. successfully 3D-printed HDPE using the FFF technique [26]. The authors solved the adhesion and warpage problems of polyethylene printing by changing the material of the build plate. The tensile properties of the 3D-printed samples were inferior but comparable with those manufactured by injection molding [26]. However, the loss of elongation at break of the FFF-printed samples with respect to the injection-molded ones indicates that further improvements of the FFF process of polyethylene are still required.

The use of carbon-based nanoparticles, such as carbon nanotubes (CNT) and graphene nanoplatelets (GNP), can improve both the processability [27] and the physical properties of polymers [28,29,30,31,32]. In particular, low concentrations of GNP or CNT increase both the complex viscosity and the storage modulus of polyethylene, act as nucleating agents, and improve the mechanical properties [33,34]. Bourque et al. [35] studied the thermal and mechanical properties of HDPE loaded with GNP nanoparticles, observing enhancements of nucleation, crystallization kinetics, and mechanical properties at all the GNP concentrations analyzed. However, tensile tests showed that small amounts of GNP (≤1 wt%) markedly improved the mechanical properties, while only marginal enhancements were obtained at higher loadings. Similarly, Chen et al. [36] investigated polyethylene loaded with graphene oxide (GO), finding that the addition of up to 1 wt% of GO enhanced both the hardness and the yield strength of polyethylene. In addition to the improvement of the mechanical performances, the interest in the use of carbon nanoparticles mainly lies in their ability to simultaneously enhance different properties. Low electrical percolation thresholds and increased thermal conductivity values are observed in polyethylene composites [37,38,39]. Carbon nanoparticles also provide electromagnetic interference shielding (EMI) that increases at increasing nanofiller concentrations [40,41]. However, while the multifunctional properties of carbon-based polyethylene composites are recognized and actively sought after, the 3D printing of polyethylene composites is still at an early stage, and very few studies can be found in the literature [30,42].

The incorporation of graphene nanoparticles into thermoplastic matrices poses several challenges due to their tendency to agglomerate, low compatibility with such polymers, and difficulties in achieving uniform dispersion [43]. In fact, nanoparticles have a high surface area-to-volume ratio, with the occurrence of strong van der Waals forces, which can induce the agglomeration. This results in non-uniform dispersion within the polymer matrix, leading to poor mechanical, thermal, and electrical properties. Moreover, low compatibility and weak interactions with the matrix result in poor adhesion between nanoparticles and the polymer, reducing the effectiveness of the reinforcement. High shear forces and temperatures may be required to achieve dispersion, which can degrade the polymer or affect the properties of the nanoparticles. To overcome these issues, several methods are employed to mix nanoparticles into thermoplastics, such as solution mixing, melt mixing, and in situ polymerization. In the case of using thermoset matrices, nanoparticles may lead to difficulties in achieving the complete polymer curing or homogeneity [44]. Moreover, the improvement of nanoparticles’ dispersion can be more difficult in thermosets due to the limited mobility of polymer chains once curing has started. This can result in localized areas of high nanoparticle concentrations, leading to variations in properties and potential performance issues.

In this work, the filament extrusion and FFF manufacturing process of composites made of medium-density polyethylene (MDPE) loaded with exfoliated graphene nanoplatelets (xGnP) were examined. Polyethylene was selected for its ability to shield against space radiation, its chemical stability, its low cost with respect to other polymers, and from the perspective of being able to recycle the PE packages used by astronauts. Graphene nanoparticles were employed as filler to enhance the mechanical, thermal, and electrical performances of the PE matrix. Therefore, the use of this reinforcement enables multifunctional properties that can be successfully exploited for space applications.

Hence, to determine the optimal percentage of graphene nanoplatelets, a screening of materials was initially conducted, studying the effects of varying graphene concentrations on the degree of crystallinity by differential scanning calorimetry (DSC). Based on these findings, filaments and 3D-printed samples for electrical measurements were fabricated using the GNP loadings of 0.5 wt% and 1 wt%. The process parameters of fused filament fabrication were optimized, and the electrical and mechanical properties of the 3D-printed composites were investigated and compared with those of molded samples, as a control. Considering the critical need to minimize charging effects in space and on extraterrestrial planets, the composites with superior electrical properties were selected (MDPE/GNP 1 wt%). Therefore, in the second part of the work, investigations were focused on the mechanical properties and behavior in high-vacuum environments of the MDPE/GNP 1 wt% composite. In particular, outgassing tests combined with solar irradiation exposure at air mass zero spectrum (AM0), which is the extraterrestrial solar irradiance at a distance of one astronomical unit, were performed to examine the in-space suitability of the 3D-printed MDPE/GNP 1 wt% samples. Moreover, a previous numerical study has demonstrated the effectiveness of MDPE/graphene composites to shield from radiation in low earth orbit (LEO), and from solar particle event (SPE) and galactic cosmic ray (GCR) radiation fields [45].

Overall, the novelty of this work relies on the use and optimization of medium-density polyethylene-based composites obtained via 3D printing for potential in-space fabrication. In fact, 3D-printed MDPE/graphene materials have not been considered for this type of application before, with respect to low-density PE composites, which are easier to print. MDPE typically exhibits better resistance to stress cracking compared to HDPE. This property is particularly advantageous in applications where the material is exposed to harsh environmental conditions, such as those experienced in space, as it reduces the risk of premature failure. In summary, this work proposes an optimized manufacturing process for the in-space 3D fabrication of composites with MDPE matrix, and their suitability in the space environment is experimentally investigated in the LARES-lab experimental facility in compliance with the European Space Agency requirements. In particular, waste materials, such as astronauts’ PE food packaging, could be reused in situ for the fabrication of multifunctional materials, such as those investigated in this study, following the optimized process parameters. This aligns with the goals of the aerospace industry in terms of sustainability and resource efficiency. This approach minimizes the need to transport bulky materials from Earth, thereby reducing mission costs and the environmental impact. By employing 3D printing technology with waste polyethylene feedstock, aerospace companies can revolutionize manufacturing processes for future space exploration missions. In fact, structural components and tools could be assembled in space.

## 2. Experimental

### 2.1. Materials

Medium-density polyethylene (product code 332119, density 0.94 g/cm^3^) was purchased from Sigma-Aldrich (Milan, Italy) in the form of a powder. Exfoliated graphene nanoplatelets of grade C750 (thickness ~2 nm, average diameter < 2 μm, SSA ~750 m^2^g^−1^) were purchased from XG Sciences (Lansing, MI, USA) and used as received. The nanoplatelets were mechanically mixed with the MDPE powder by an overhead stirrer (BS Overhead Stirrer, Velp Scientifica, Usmate Velate, Italy) for 15 min, setting a motor speed of 50 rpm. The mixed powders were then used to create pellets of MDPE/xGnP composites at filler loadings of 0.5 wt% and 1 wt% (with respect to the polymer mass). Pellets were prepared by melting in an oven at 125 °C while degassing, followed by cooling and breaking into small pieces. The dispersion of GNP into the MDPE matrix was achieved during the filament extrusion process. In fact, during this process, GNP agglomerates were subjected to shear stresses that promoted their breakage and exfoliation, improving the dispersion into the polymer. Based on the DSC results discussed in Section 3.3, in particular regarding the degree of crystallinity, the filler contents of 0.5 wt % and 1 wt% were selected as the most promising for obtaining 3D-printed samples with good mechanical properties. Extruded filaments were fabricated from the pellets using a FilaFab Pro 350 Ex extruder (D3D Innovations Limited, Lytchett Matravers, Poole, UK) with a 3 mm diameter nozzle.

**Figure 1:**
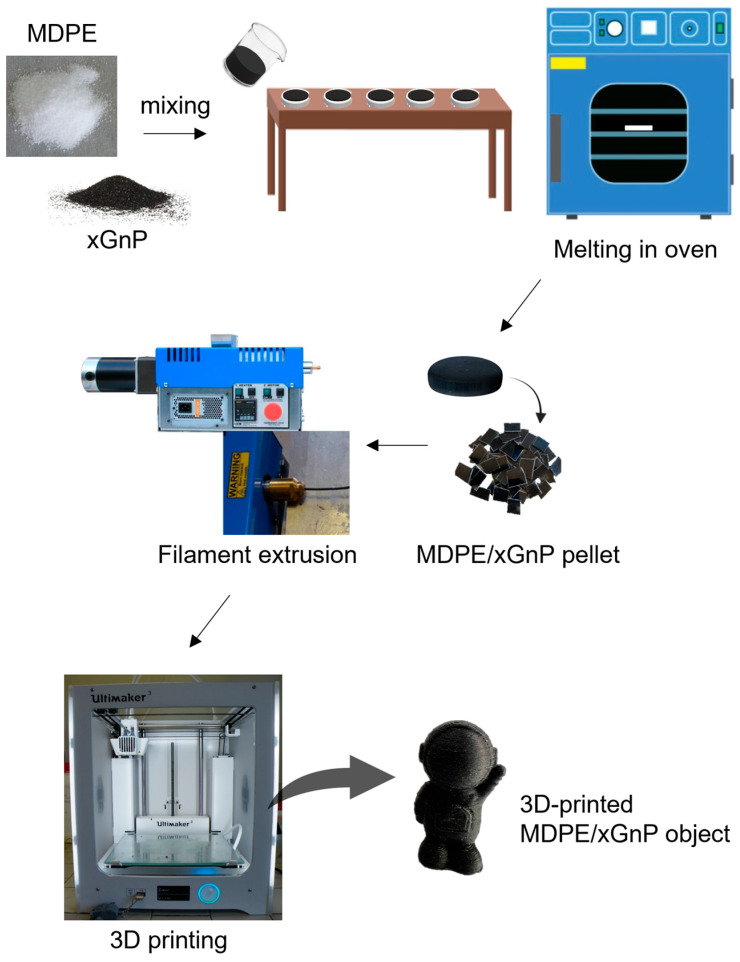
Schematic representation of pellet preparation, filament extrusion, and 3D printing of MDPE/xGnP samples.

Samples for electrical and mechanical characterization were prepared by FFF using a Ultimaker 3 printer (Ultimaker B.V., Utrecht, The Netherlands) with a 0.8 mm diameter nozzle. This diameter was chosen over 0.4 mm to avoid nozzle scratching and clogging caused by the presence of carbon nanoparticles [46]. Moreover, the use of a 0.8 mm nozzle for extruding PE-graphene samples has been shown to have several advantages over a 0.4 mm nozzle [26]. In particular, a lower extrusion temperature can be employed, and the resulting filaments have higher values of Young’s modulus and tensile strength than those obtained using the 0.4 mm nozzle. In addition, using a lower extrusion temperature allows for less shrinkage and warpage during the solidification phase on the build plate. The Ultimaker Cura 4.1.0 software was used to generate the GCode files required for the FFF printer based on CAD models. For comparison purposes, samples were also prepared via the casting process, melting the composite powders in a silicon mold at 125 °C while degassing. A schematic representation of pellet preparation, filament extrusion, and 3D printing of MDPE/xGnP samples is reported in Figure 1.

### 2.2. Characterization Methods

Thermal analysis was performed on MDPE and MDPE/xGnP composite powders using a double-furnace differential scanning calorimeter (DSC 8500, PerkinElmer, Waltham, MA, USA). DSC measurements were carried out in the temperature range from −45 °C to 150 °C with heating and cooling rates of 10 °C/min.

**Figure 2:**
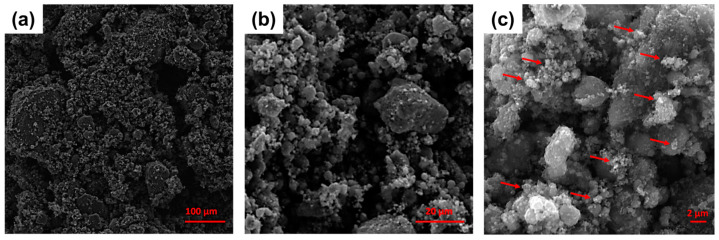
SEM images of the as-received xGnP powder at magnifications of (**a**) 500×, (**b**) 3000×, and (**c**) 10,000×. Red arrows are used to indicate the nanoparticles (diameter < 2 µm, from XG Sciences datasheet).

Scanning electron microscopy (SEM) investigations were carried out using a VEGA II LSH instrument (Tescan, Brno, Czech Republic). The xGnP powder was imaged as received at an accelerating voltage of 10 kV and different magnifications (Figure 2). The morphology of filaments and 3D-printed samples was evaluated at different magnifications using an accelerating voltage of 5 kV. Before SEM imaging, samples were coated with gold using the Cressington 108 Auto Sputter Coater (Cressington Scientific Instruments, Watford, UK).

Raman investigations were performed using a micro-Raman XploRA Plus system (Horiba Scientific, Lille, France) equipped with a confocal optical microscope. Analyses were carried out on the surface of the extruded filaments after optimization of the extrusion process. Measurements were performed with a 532 nm excitation wavelength, 10× microscope objective, and adopting a diffraction grating of 600 g/mm. A filter of 10% was used to avoid heating and damaging of the filament surface during investigation. Raman maps were acquired by scanning a sample surface up to 600 μm × 600 μm with a step size of 7 μm in both the X and Y axes. Raman maps were obtained by plotting the relative intensity or the position of the peaks associated with MDPE and graphene, which were identified in the corresponding Raman spectra for their relevance. Maps were processed using the Horiba LabSpec 6 software.

**Figure 3:**
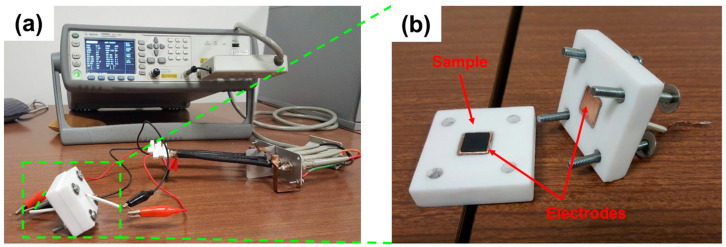
Setup for electrical measurements: (**a**) LCR instrument used for the electrical measurements and (**b**) specimen (10 mm × 10 mm × 1.5 mm) placed in the custom-made Teflon cell and contacted by means of flat copper electrodes on the top and bottom surfaces.

The AC electrical impedance (Z) of molded and 3D-printed samples of MDPE and MDPE/xGnP at 0.5 and 1 wt% was determined over the frequency range of 1–2 MHz using an E4980A Precision LCR Meter (Agilent, Santa Clara, CA, USA). The specimens (10 mm × 10 mm × 1.5 mm) were placed in a custom-made Teflon cell and contacted by means of flat copper electrodes on the top and bottom sample surfaces (Figure 3). The impedance data were averaged over at least 20 measurements, and the mean value was normalized by the sample thickness (Z_normalized_ = Z/t, t = 1.5 mm).

The tensile strength of molded and 3D-printed samples of MDPE and MDPE/xGnP at 1 wt% was measured with a 3382 dual-column floor model Universal Testing System (Instron, Norwood, MA, USA) according to DIN EN ISO 527-2 standard [47]. A load speed of 0.5 mm·min^−1^ was used, and the tensile strength values were averaged over five sample measurements.

Surface wettability tests were carried out measuring the contact angles of water droplets on the sample surface using an optical analyzer (OCA15Pro, DataPhysics Instruments, Filderstadt, Germany). The static sessile method (droplet volume 3 μL) was selected, and ultrapure water (Milli-Q, 18.2 MΩ cm) and diiodomethane were used as testing liquids. Ten droplets on different areas of each sample were analyzed. Contact angle values were obtained by drop shape analysis using the DataPhysics SCA 20 software module. The surface free energies (SFE) were calculated using the contact angle values measured with water and diiodomethane, following the Owens–Wendt method [48]:

γ_l_ (1 + cosθ) = 2 [(γ_s_^d^ γ_l_^d^)^1/2^ + (γ_s_^p^ γ_l_^p^)^1/2^] (1)

where γ_s_ is the SFE of the investigated sample, γ_l_ is the SFE of the measuring liquid, the apexes d and p indicate the dispersive and polar components, respectively, and θ is the contact angle between the sample and the testing liquid.

### 2.3. Experimental Setup for Tests in Simulated Space Environment

**Figure 4:**
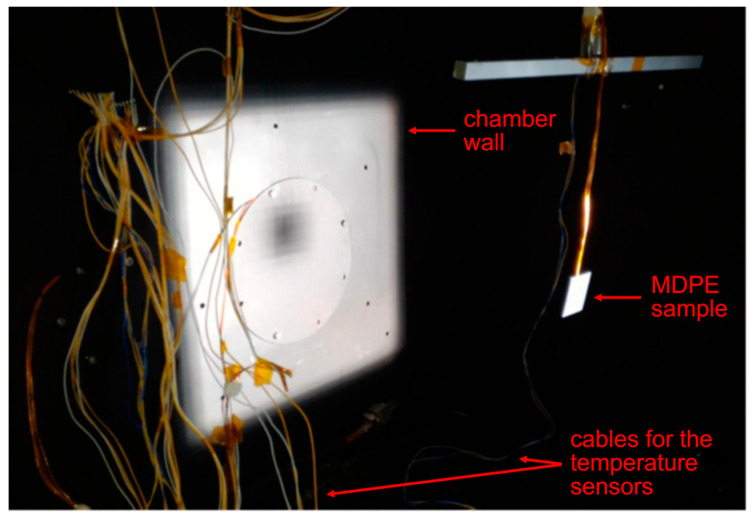
Neat MDPE sample placed at the center of the AM0-spectrum beam in the thermo-vacuum chamber.

The 3D-printed samples of MDPE and MDPE/xGnP at 1 wt% were tested in a simulated space environment [49,50] using the LARES-lab experimental facility, which complied with the European Space Agency standard requirements (ECSS, 2012; ECSS, 2002) [51]. The thermo-vacuum chamber has an internal dimension of 60 × 60 × 60 cm^3^ and is characterized by walls entirely covered by a copper shroud painted with Aeroglaze Z306—a vacuum-compatible black paint with high absorptivity and emissivity (ε = 0.89, α = 0.97) [52]. The pressure was maintained below 10^−6^ mbar using two pumps: a XDS5 dry scroll pump and a EXT255DX turbomolecular pump (Edwards Vacuum, Burgess Hill, West Sussex, UK). The first one brings the pressure below 2 mbar, the limit required to start the second pump, which brings the pressure to the operational value. The turbomolecular pump and the pressure gauge communicate with a controller connected to a personal computer and software (Edwards TIC PC Program, version 2.0.0) to monitor the pressure and the pump parameters. The cooling shroud was composed of five copper plates cooled by an open-circuit liquid nitrogen coil. Solar radiation was simulated by a SpectroSun XT-10 (Spectrolab, Sylmar, CA, USA) provided by an OSRAM XBO 1000-W lamp (OSRAM, Munich, Germany) that projects a AM0-spectrum beam with a constant power over a 12 × 12 cm^2^ surface. The temperatures were recorded by platinum resistance thermometers PT100, and two monitoring systems allowed for recording data from up to 12 PT100 sensors. The neat MDPE and composite samples were tested one at a time and preconditioned at 23 °C and RH 50% for 24 h before testing. Each sample was weighted immediately before the test and positioned in the chamber, as shown in Figure 4, setting a starting temperature of 22 °C. The vacuum was applied, and the solar radiation simulator was started when the turbomolecular pump assured the minimum pressure (≤10^−6^ mbar). The solar irradiation was set for 4 h, and for the remaining time only the vacuum was maintained. The entire duration of the test was 24 h. At the end of the test, the vacuum was switched off and the chamber was brought to the initial temperature (22 °C). Samples were immediately weighted after removal from the chamber.

The samples were then post-conditioned in 50% relative humidity and ambient atmosphere for 24 h. After post-conditioning, the samples were weighed to calculate the recovered mass loss percentage (RML%) and the water vapor regained percentage (WVR%). The percentages of total mass loss percentage (TML%), the RML%, and the WVR% were calculated as indicated by the ASTM E595 standard [53] according to the following expressions:

(2) $$TML\%=\frac{M_{0}-M_{f}}{M_{0}}\times 100$$

(3) $$WVR\%=\frac{M_{f}^{\prime }-M_{f}}{M_{0}}\times 100$$

(4) $$RML\%=\frac{{M_{0}-M}_{f}^{\prime }}{M_{0}}\times 100$$

where M_0_ is the initial mass of the specimen, M_f_ is the final mass, and $M_{f}^{\prime }$ is the final mass measured after the reconditioning step at room temperature.

## 3. Results and Discussion

### 3.1. Optimization of the Filament Fabrication

The quality of the filament, specifically its dimensional precision and cross-sectional roundness, plays a crucial role in preventing failures in the 3D printing process and ensuring the production of high-quality parts. The filament is guided through the feeder into the print head based on factors such as material density and filament diameter, which are considered constant throughout the printing process. As a result, the volume of the 3D-printed material relies entirely on the cross-sectional area of the filament and the printing speed [54]. Inconsistencies in the filament diameter along its length can lead to a poor surface finish and a decline in the quality of printed products. This is because the 3D printer controller does not account for such variations. Therefore, it becomes essential to meticulously select the parameters of the filament fabrication process, including the chamber temperature and screw speed. To optimize these parameters, both were systematically adjusted until uniform and smooth filaments were achieved. For each type of material, whether it was neat MDPE or MDPE/xGnP composites, the initial chamber temperature was determined based on the melting temperature of the powders, as determined through thermal analysis (Table 1). This temperature was then increased by 10 °C to ensure complete material melting in the chamber and subsequently reduced, while the screw speed was incrementally raised.

**Figure 5:**
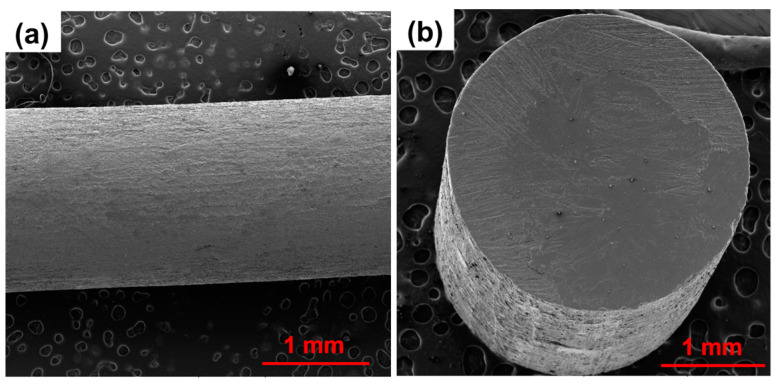
SEM images of the (**a**) longitudinal and (**b**) transversal view of the extruded filament of the MDPE/xGnP 1 wt% composite at 50× and 80× magnifications, respectively.

During each experiment, once the chamber temperature was reached, a 20 min delay was observed to facilitate uniform heating of the material and prevent any machine damage. After numerous tests, it was determined that the optimal chamber temperature for both neat MDPE and MDPE/xGnP composites at 0.5 wt% and 1 wt% was 118 °C. This careful selection and fine-tuning of parameters were essential to produce high-quality filaments for the 3D printing process. It was determined that the optimal screw speed was 5 rpm, corresponding to a filament extrusion speed of approximately 2 cm per minute. Using these parameters, filaments of MDPE and MDPE/xGnP composites with good surface quality and an average diameter of 2.87 ± 0.02 mm were successfully fabricated. For instance, in Figure 5, the SEM images depict both the longitudinal and transversal views of an extruded filament of the MDPE/xGnP 1 wt% composite. These optimized extruded filaments demonstrate a round cross-sectional area and show no internal defects or surface irregularities. It is worth noting that the optimized chamber temperature was slightly higher than the melting temperature determined by dynamic differential scanning calorimetry (as shown in Table 1) and in the datasheet of neat medium-density polyethylene (~110 °C). This temperature adjustment is necessary to compensate for the conductive and convective heat dissipation that occurs during the extrusion process.

**Figure 6:**
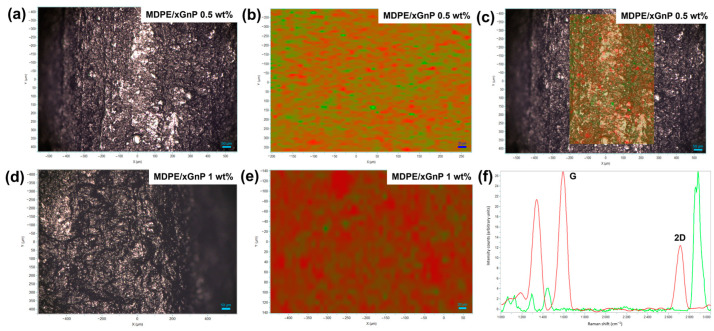
(**a**,**d**) Confocal optical images, (**b**,**e**) Raman maps, and (**c**) merged image from confocal microscopy and Raman mapping of the surface of MDPE/xGnP filaments at 0.5 wt% and 1 wt% of filler loading. (**f**) Raman spectra of xGnP particles (red curve) and MDPE (green curve).

Raman mapping was performed on the surface of the MDPE/xGnP filaments at 0.5 and 1 wt%, combined with the image acquisition of the investigated areas by confocal optical microscope (Figure 6). The Raman spectra acquired for each point of the prefixed grid were expressed as a linear combination of spectra of pure MDPE and xGnP powders (Figure 6f). The values of the coefficient of linear combination were plotted (Figure 6b,e) in a color scale, in which red areas represent the surface regions where the xGnP particle content was higher. In both cases, filaments at 0.5 and 1 wt%, a good dispersion of the graphene filler was observed. Supplementary evidence for the exfoliation of graphene can be inferred from a peak analysis of the Raman spectra in Figure 6f, in which the red curve refers to the Raman signal intensity of graphene. The peak at about 2700 cm^−1^ (2D band) is sensitive to the number of graphene layers and tended to decrease significantly with the increasing number of layers [55]. In particular, the ratio between the intensities of the peaks at 2700 cm^−1^ (2D band) and at 1600 cm^−1^ (G band), which is between 0.4 and 0.5 (I_2D_/I_G_ = 0.46), suggests the formation of few layers (4–5) of graphene [56]. This is in agreement with the thickness (~2 nm) of the nanoplatelets of grade C750 used in this work, as measured by the producer (XG Sciences).

**Figure 7:**
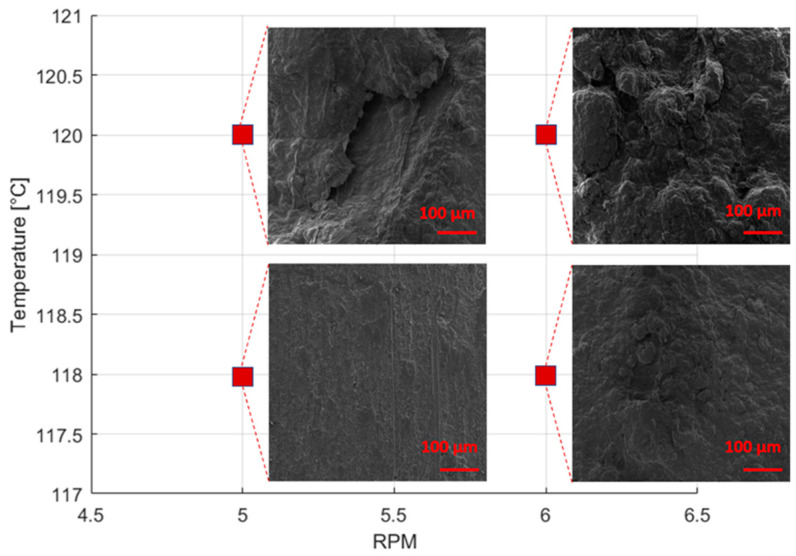
SEM images (500×) of the longitudinal view of the extruded MDPE/xGnP 1 wt% filament at different extrusion temperatures and screw speeds.

Any attempt to increase the temperature or screw speed beyond 118 °C and 5 rpm, respectively, led to inconsistent filament diameters or the production of molten filaments across all graphene loadings, as illustrated in Figure 7. Conversely, reducing the temperature resulted in the creation of extremely fragile filaments, marked by non-melted areas, or filaments with larger diameters (measuring 2.92 ± 0.02 mm at 117 °C and 5 rpm). Furthermore, it is noteworthy that the optimized process parameters were consistent for filaments made of neat MDPE and those composed of MDPE/xGnP composites. This underscores the predominance of the behavior of the polymer matrix over the presence of graphene, at least within the range of filler concentrations examined in this study. However, the presence of graphene nanoplatelets had a substantial influence on the 3D printing parameters of MDPE, as shown in the following section.

### 3.2. Optimization of the 3D Printing Process

Polyethylene is renowned for its challenges when it comes to 3D printing. It presents significant adhesion and warping issues that can lead to unsuccessful print jobs. Warpage, especially prominent in semi-crystalline thermoplastic materials, such as polyethylene [57], occurs due to the distortion resulting from non-uniform shrinkage caused by uneven cooling during the manufacturing process.

Adhesion is fundamentally influenced by the degree of contact between surfaces. Therefore, a polymer with high surface energy is preferable as it tends to exhibit strong adhesion forces and facilitates the complete wetting of the polymer onto the substrate. Wetting occurs when there is a minimal difference between the surface energies of the materials in contact [58]. However, being a non-polar polymer, polyethylene is characterized by an extremely low surface energy (as low as 20 mJ/m^2^) that prevents the material from adhering to common 3D printing substrates [26]. The analysis of the surface wettability by the static contact angle unveiled higher hydrophobicity for the MDPE/xGnP 1 wt% samples with respect to neat MDPE (Table 2). Moreover, composites showed higher values of surface free energy, and thus potentially better adhesion with the build plate.

In this work, different approaches were adopted to overcome the adhesion issues of MDPE and to 3D-print parts with tight dimensional tolerances. Initially, a glass build plate was used. However, even after increasing the temperature of the glass build plate to 100 °C or using brims or rafts, detachment of the samples occurred, accentuated by the polymer warpage. This problem was only partially solved by using a double-sided adhesive tape. With this solution, it was possible to print square samples of MDPE (10 mm × 10 mm × 1.5 mm) with the help of a brim or a raft. The parameters used are listed in Table 3.

**Figure 8:**
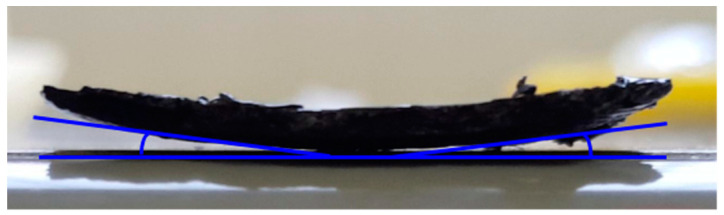
Warpage phenomenon for the 3D-printed specimen of the MDPE/xGnP 1 wt% composite.

The unsuitable build plate material and the low working temperature (70 °C) did not permit the realization of larger MDPE or MDPE/xGnP composite samples, which would detach from the build plate due to a combination of poor adhesion and warpage (Figure 8).

**Figure 9:**
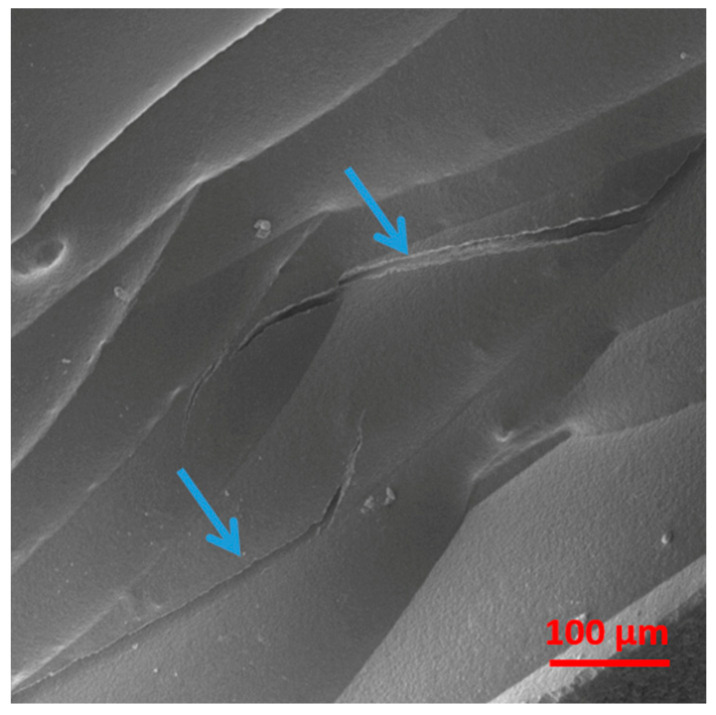
SEM image of layers’ delamination, indicated by arrows, on a 3D-printed specimen of the MDPE/xGnP 1 wt% composite at 500× magnification.

These phenomena also generated delamination of the deposited layers, as a consequence of sample bending, as shown in Figure 9 for the MDPE/xGnP 1 wt% composite. For the square samples, the only pattern that enabled the realization of a 100% infill density was the concentric pattern, whereas the tensile samples were best printed with a linear infill pattern.

**Figure 10:**
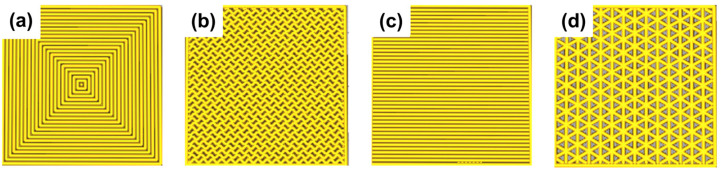
Examples of different infill patterns: (**a**) concentric, (**b**) gyroid, (**c**) lines, and (**d**) triangles.

Other infill patterns, such as triangles and gyroids (Figure 10), resulted in printing failures or low printing qualities. The 100% infill density was used for all types of 3D-printed specimens, whose electrical and mechanical properties were measured and compared with those of equivalent molded specimens.

The warpage and adhesion problems associated with the 3D printing of MDPE-based materials were solved using a HDPE build plate instead of the glass one. Using a HDPE substrate, it was possible to print complex samples due to the low interfacial tension between MDPE and the HDPE build plate, which translates to high adhesion strength [58,59]. In this case, a careful setting of the printing parameters, mostly the build plate temperature, is essential to prevent the welding of the printed material on the HDPE build plate [59,60].

**Figure 11:**
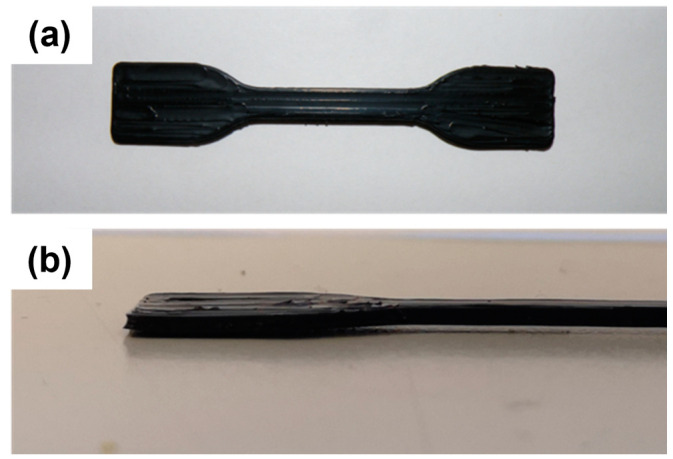
(**a**) Top and (**b**) lateral views of the tensile specimen (DIN EN ISO 527-2-5A) of the MDPE/xGnP 1 wt% composite fabricated by 3D printing on the HDPE build plate.

Square samples of MDPE and MDPE/xGnP composites at 0.5 and 1 wt% filler loadings were 3D-printed for electrical impedance measurements, and their properties were compared with those of molded samples. Similarly, specimens of MDPE and MDPE/xGnP 1 wt% were printed for tensile tests, with their geometry defined according to DIN EN ISO 527-2 (5A) [47] (Figure 11). Samples of MDPE containing more than 1 wt% of graphene were intentionally excluded from the printing process to prevent nozzle wear [46]. Additionally, this precaution aimed to avoid other undesirable phenomena, such as deep bed filtration or cake filtration, which have the potential to alter the final properties of the printed part and, in extreme cases, lead to nozzle blockages.

The tensile specimens were printed using a symmetric and balanced linear infill pattern ([0°/90°]_s_). This approach alternated the 0° and 90° infill pathways, aiming to minimize residual stresses [61], while preserving the high tensile properties of the printed samples. The optimized 3D printing parameters are summarized in Table 4.

A different build plate temperature was used to print the neat MDPE samples and the MDPE composite samples. It was observed that a HDPE build plate temperature of 60 °C enabled the printing of neat MDPE parts but resulted in printing failures when using filaments of MDPE/xGnP with graphene loadings of 0.5 wt% or 1 wt%. The printing of MDPE composites required an increase of the HDPE build plate to 80 °C and keeping the printing chamber at 40 °C. Lower temperatures did not facilitate proper adhesion, while higher temperatures led to welding and subsequently made it challenging to detach the specimen from the build plate. The incorporation of graphene nanoparticles or carbon nanotubes as fillers is known to enhance the thermal stability of the polymer, thereby reducing issues related to warpage and shrinkage [31], and improving the quality of the printed part. However, it is noted that xGnP, being a filler made of highly pure carbon, possesses a strong non-polar character and higher surface energy compared to polyethylene [62]. This incorporation of the xGnP nanofiller altered the surface energy and polarity of the MDPE matrix, leading to an increase in interfacial tension between the printed material and the HDPE substrate. Consequently, this change in properties resulted in decreased adhesion strength. Nonetheless, despite the use of xGnP, careful adjustment of the parameters allowed for the successful 3D printing of composite samples for subsequent electrical and tensile characterization. This meticulous parameter tuning was crucial in preventing printing failures caused by incompatible settings.

**Figure 12:**
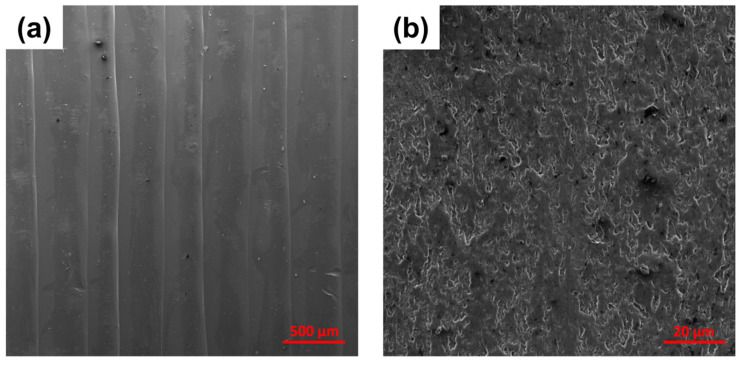
SEM images of (**a**) surface and (**b**) cross-sectional area of 3D-printed MDPE/xGnP 1 wt% samples fabricated for tensile tests.

Figure 12 shows SEM images of the surface and cross-sectional area of the 3D-printed MDPE/xGnP 1 wt% samples fabricated for tensile tests. In Figure 12a, the alignment of the printed filaments can be clearly observed. The cross-sectional area (Figure 12b) shows a compact structure with a small number of voids or defects.

### 3.3. Thermal Analysis by Differential Scanning Calorimetry

**Figure 13:**
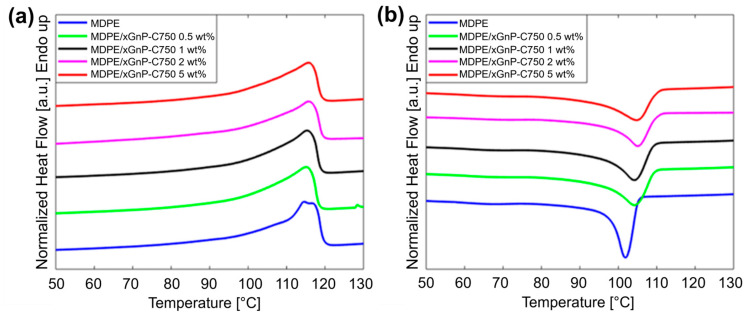
DSC thermograms during (**a**) heating and (**b**) cooling of neat MDPE and MDPE/xGnP composite powders at 10 °C/min. Data are offset for clarity.

The concentration and the dispersion state of the nanoparticles and their interfacial bonding with the polymer matrix affect the physical properties of the overall composite material, including its melting and crystallization behavior. For this reason, prior to 3D printing, thermal analysis by DSC was performed on the neat MDPE and on the MDPE/xGnP composite powders with different concentrations of graphene nanoparticles. The effect of the nanoparticles on the non-isothermal melting and crystallization behavior of the powders was assessed in order to correctly set the temperature of the extrusion and FFF processes. Additionally, the crystallinity of the printed materials was assessed, as this factor can have a substantial impact on the mechanical properties of the 3D-printed composites. Figure 13 reports the DSC curves during heating and cooling of neat MDPE and of MDPE/xGnP composite powders with 0.5, 1, 2, and 5 wt% of graphene filler. The results from the thermal analysis are summarized in Table 1.

All composites showed similar melting temperatures (T_m_). The limited role of carbon nanoparticles on the composites T_m_ was also reported by other authors [63]. Regarding the melting enthalpy (ΔH_m_), its value was approximately the same for composites at 0.5 and 1 wt% of filler as for neat MDPE, and then decreased at higher graphene loadings (2 wt% and 5 wt%). Contrary to the melting temperature, the crystallization temperature (T_c_) of the composites was more affected by the presence of graphene nanoparticles. The T_c_ of neat MDPE was 102 °C and increased to 104 °C for the MDPE/xGnP composites at 0.5 and 1 wt% filler loadings, and to 105 °C for the MDPE/xGnP at 2 and 5 wt%. Although the increase of T_c_ was observed at all graphene loadings, the largest increase occurred at the lower nanofiller content. The increase of T_c_ and the stability of T_m_ translate to lower degrees of supercooling (ΔT_s_ = T_m_ − T_c_) for the MDPE/xGnP composites with respect to the neat MDPE, indicating that the total crystallization time decreased with the addition of graphene nanoparticles. In addition, when a polymer crystallizes with less supercooling, it crystallizes more perfectly, and the crystallinity is expected to be enhanced [64]. The increase in T_c_ along with the low degree of supercooling of MDPE/xGnP composites suggest that the graphene filler acts as a nucleating agent, inducing the formation of heterogeneous nuclei [65] and enhancing the crystallization of the polymer. The degree of crystallinity (χ) was evaluated from the melting enthalpies, using the following expression:

(5) $$\chi ={∆H}_{m}/{[∆H}_{f}\left(1-w_{f}\right)]$$

where ΔH_f_ is the latent heat of fusion of 100% crystalline polyethylene (288 J/g) [66] and w_f_ is the weight fraction of the nanoparticles. As shown in Table 1, the crystallinity slightly increased at 0.5 and 1 wt% of xGnP in comparison with the neat MDPE and decreased at 2 and 5 wt% of xGnP. The reduction in crystallinity observed in the composites with 2 wt% and 5 wt% of graphene suggests that while nanoparticles do serve as nucleating agents, the abundance of nucleating sites at higher filler contents restricts the mobility of polymer chains. This limitation, in turn, hinders the formation of crystalline lamellae. Furthermore, the decreased mobility of polymer chains is also influenced by the substantial number of graphene aggregates that tend to form at higher filler concentrations.

**Figure 14:**
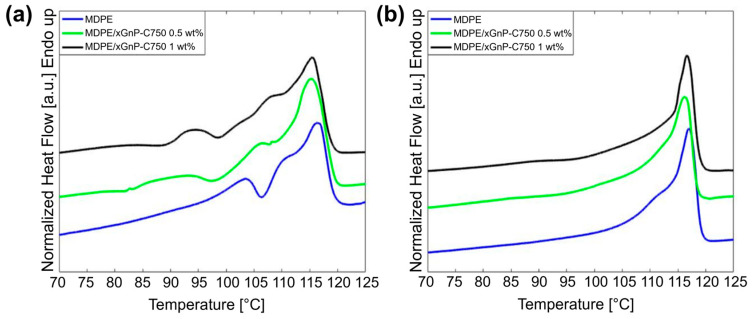
DSC thermograms during heating of MDPE and MDPE/xGnP composites at 0.5 and 1 wt% filler loading in the form of (**a**) extruded filaments and (**b**) 3D-printed samples. Heating rate: 10 °C/min. Data are offset for clarity.

In consideration of the DSC measurement results and to prevent nozzle wear or clogging, MDPE composite filaments and 3D-printed samples were produced with xGnP loadings of 0.5 wt% and 1 wt%. These samples exhibited higher crystallinity (Table 1), proving the most promising for obtaining 3D-printed composites with good mechanical properties. Consequently, higher percentages of GNP were disregarded, and thus filaments and the samples for electrical measurements were fabricated using 0.5 wt% and 1 wt% GNPs. To evaluate the impact of the manufacturing process on the crystallinity of the 3D-printed parts, DSC analysis was performed on both the filaments and the 3D-printed samples of MDPE and MDPE/xGnP at 0.5 wt% and 1 wt% filler loadings (Figure 14a,b). As shown in Figure 14a, the DSC melting curves of the filaments exhibited three distinct endothermic peaks. The presence of multiple peaks is indicative of high crystallization rates and is attributed to the melting of imperfect crystals formed during the cooling phase [67,68]. In this study, the extruded filaments experienced rapid cooling, which naturally led to the presence of multiple peaks in the DSC analysis due to imperfect crystal growth [69]. The smaller endothermic peak observed at a lower temperature can be attributed to the melting of defective crystals, indicating their presence within the material. Conversely, the larger peak corresponds to the melting of primary crystals that form during the initial crystallization process, involving primary nucleation and crystal growth. Additionally, the middle shoulder in the DSC curve is linked to the melting of secondarily crystallized material. This material is typically expelled from the primary lamellae and is expected to melt first, often in a step-like manner [70].

Unlike the filaments, the 3D-printed samples exhibited one endothermic peak, indicating that the cooling rate of each layer as it was deposited onto the heated build plate was lower. The melting enthalpies for the 3D-printed samples of MDPE and MDPE/xGnP at 0.5 wt% and 1 wt% filler concentrations were measured at 106.1 J/g, 108.1 J/g, and 111.0 J/g, corresponding to crystallinity degrees of 36.8%, 37.7%, and 38.9%, respectively. The crystallinity of the 3D-printed samples was marginally lower than that of the corresponding powders (Table 1). This discrepancy can likely be attributed to the uncontrolled cooling of the material during the printing process. It is worth highlighting that the thermal history associated with the filament extrusion process did not appear to have a significant impact on the thermal behavior of the 3D-printed parts. This was evident from the absence of multiple peaks in the DSC thermograms of the printed parts, as shown in Figure 14b.

### 3.4. Electrical Properties

The electrical properties of MDPE and MDPE/xGnP composites containing 0.5 wt% and 1 wt% of filler were investigated using electrical impedance spectroscopy (EIS) within the frequency range of 1 kHz to 2 MHz. Since these materials exhibit substantial impedance values at lower frequencies, EIS data were collected exclusively at frequencies exceeding 1 kHz. This decision was made to ensure the accuracy of the measured impedance values, particularly near the instrument’s measurement limit. In the frequency range of 1 kHz to 2 MHz, the electrical impedance curves demonstrated a linear trend. This linear behavior allowed for a detailed examination of the impact of various manufacturing techniques on the electrical properties of the composite.

**Figure 15:**
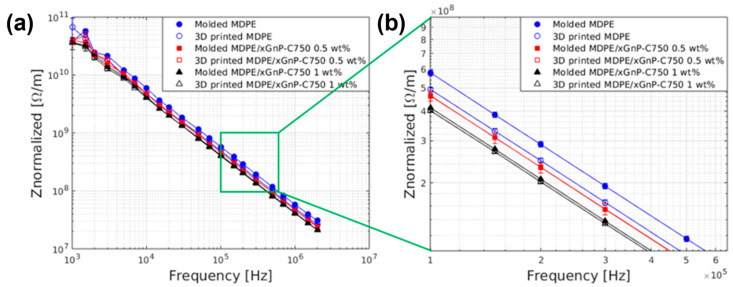
(**a**) Bode plot with normalized impedance (Z_normalized_) of molded and 3D-printed MDPE and MDPE/xGNP composite samples at 0.5 and 1 wt% filler loading (square specimens 10 mm × 10 mm). (**b**) Enlarged area of the Bode plot in the impedance range 10^8^–10^9^ Ω/m and in the frequency range 1 × 10^5^–6×10^5^ Hz. Standard deviation of data was below 2%.

All reported impedance data were normalized by the sample thickness. As shown in Figure 15, the mean values of the normalized impedance (Z_normalized_) for both 3D-printed and molded samples (10 mm × 10 mm × 1.5 mm) exhibited a linear decrease with the increasing frequency, a characteristic trend of dielectric materials. It is noteworthy that the electrical behavior of molded and 3D-printed samples was similar, implying that the 3D-printed samples did not have defects, such as voids, and the 3D printing process did not significantly alter the electrical properties of the composite materials. Additionally, the 3D-printed samples of neat MDPE exhibited a lower Z_normalized_ value compared to the molded ones. This difference may be attributed to the increased permittivity of the 3D-printed parts at the interface between adjacent deposited layers [59]. This phenomenon resulted in a slight but noticeable reduction in overall electrical impedance due to the heightened capacitance. However, such an effect was not observed in the composite specimens, likely due to the decrease in resistance caused by the presence of graphene.

Comparing the impendence values, a 33% decrease in impedance for MDPE/GNP 1 wt% and a 20% decrease for MDPE/GNP 0.5 wt% were observed with respect to pristine MDPE. This suggests that the addition of GNP may enhance the material’s ability to manage electric charges, thereby reducing the charging effects and improving its overall electrical conductivity. Considering the critical need to minimize charging effects in space and on extraterrestrial soils, materials with reduced resistivity are preferable to ones that are more insulating. From this point of view, MDPE/GNP 1 wt% is more advisable with respect to MDPE/GNP 0.5 wt% and pristine MDPE for use in future long-term human exploration missions.

### 3.5. Mechanical Properties

The results of the electrical impedance spectroscopy highlighted how the inclusion of GNP at 1 wt% significantly enhanced the overall electrical properties of the MDPE polymer compared to 0.5 wt%. For this reason, the focus of the mechanical properties and outgassing characteristics was on MDPE/GNP 1 wt% material.

**Figure 16:**
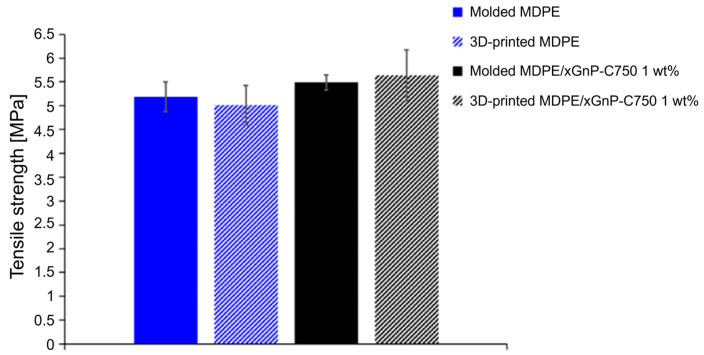
Tensile strength of molded and 3D-printed MDPE and MDPE/xGnP 1 wt% specimens (DIN EN ISO 527-2-5A).

Using the optimized 3D printing parameters, tensile specimens of MDPE and MDPE/xGnP at 1 wt% were 3D-printed for testing according to DIN EN ISO 527-2-5A [47]. The tensile strength of the 3D-printed specimens was measured and compared to that of molded specimens (Figure 16). Mean values of the tensile strength were 5.19 ± 0.31 MPa for the molded MDPE and 5.01 ± 0.42 MPa for 3D-printed MDPE, and 5.49 ± 0.16 and 5.64 ± 0.53 for the molded and the 3D-printed MDPE/xGnP composite at 1 wt%. In the case of molded samples, there was an approximate 5.5% difference in tensile strength between MDPE (5.19 ± 0.31 MPa) and MDPE/xGnP at 1 wt% (5.49 ± 0.16 MPa). On the other hand, for the 3D-printed samples, there was an increase of about 12.6% for the tensile strength of MDPE/GNP 1 wt% with respect to pristine MDPE. This higher tensile strength in the molded composite can be attributed to the effective stress transfer between the polymer matrix and the nanofiller.

These findings align with results from other studies conducted by different authors [34,35], which also observed comparable improvements in the tensile strength of HDPE with the incorporation of GNP within the range of 0.5 wt% to 1.5 wt%. A recent study utilized selective laser sintering to 3D-print HDPE tensile specimens [71]. However, the tensile strength of laser-sintered HDPE specimens was significantly lower than that achieved with traditional manufacturing techniques [34,35]. In this study, both the 3D-printed and molded specimens exhibited similar mechanical properties, underscoring the effectiveness of the FFF process and the relevance of the optimized process parameters. In fact, considering the standard deviations, the tensile strength of the 3D-printed and molded samples are comparable. This has already been observed in the literature [26], indicating a high filling degree of the 3D-printed parts. The selection of the infill pattern ([0°/90°]_s_) was anticipated to yield comparable tensile strength between the 3D-printed and molded specimens, and the results validated this expectation. Simultaneously, it was noticeable that the standard deviation of the tensile strength for the 3D-printed MDPE and MDPE/xGnP materials at 1 wt% was slightly higher when compared to the standard deviation of the molded ones. This difference can be attributed to the defects inevitably introduced by the FFF process. These defects include thermal stresses induced by the uncontrolled cooling of the molten filament during printing, which can lead to sample warpage and layers’ delamination. Additionally, as the filaments were deposited adjacent to each other, gaps between them may occur. All these factors can contribute to the fabrication of 3D-printed hardware with properties that are less strictly reproducible. However, this variability is not a critical concern for maintenance tools and utensils during manned space missions, especially considering that they can be easily recycled using the proposed technology.

### 3.6. Tests in Simulated Space Environment

Depending on the specific mission, spacecraft materials must be able to withstand various space environmental factors, including exposure to high-vacuum conditions, solar UV radiation, atomic oxygen, and ionizing radiations. The fundamental test to qualify material suitability for any space applications is the material exposure at vacuum conditions and high temperature, typically 125 °C, to evaluate the outgassing properties. Indeed, the outgassed material is detrimental not only for the degraded material itself but also for increasing the molecular contamination surfaces. Such contamination alters the surface properties of the other external satellite components, foremost the thermo-optical ones, or interferes with their functionality, such as in the case of optical sensors. Furthermore, the combined effects of vacuum and ultraviolet (UV) radiation can enhance outgassing in polymers, as UV-C radiation is energetic enough to break molecular bonds. As a result, the 3D-printed composite material was tested in a simulated space environment, which involved exposure to vacuum conditions along with solar irradiation.

Table 5 provides an assessment of the outgassing properties of the 3D-printed MDPE/xGnP 1 wt% and MDPE samples. Both types of materials exhibited total mass loss (TML%) and recovered mass loss (RML%) values of less than 1, indicating that they are suitable for space applications, in accordance with the ASTM E595 standard [53]. Notably, the total mass loss of the composite material was approximately an order of magnitude greater than that of neat MDPE, although it still remained well below the 1% threshold. This difference in mass loss could be attributed to the presence of graphene nanoplatelets, which might interfere with the polymer chain bonds. Additionally, xGnP can alter the thermo-optical properties of MDPE, resulting in an increase in the equilibrium temperature (T_e_), which is the maximum temperature measured at the back surface of the sample under irradiation. This is due to the much higher thermal conductivity of GNP [72], as compared to that of the polymer. A higher T_e_ indicates that the composite material might be advantageous, as compared to the neat polymer, in those applications in which heat dissipation is crucial. As for water vapor regain (WVR%), the ASTM standard does not specify a limited value. However, it is evident that both neat MDPE and MDPE/xGnP 1 wt% exhibited a very low tendency to absorb water vapor.

## 4. Conclusions

This study highlighted the potential of polyethylene/graphene composites processed with FFF for supporting long-term human space missions. By utilizing waste materials, such as astronaut food packaging made from polyethylene, it is possible to 3D-print tools and objects needed during space journeys and on lunar or Martian bases. These polymeric structures can be designed for specific functions and assembly, reducing manufacturing, assembly, integration, and testing (MAIT) resources and the development time. In addition, incorporating nanoparticles into the polymer matrix enhanced the space environmental compatibility of the components. To determine the optimal percentage of graphene nanoplatelets, a screening of materials was initially conducted. Based on the results in terms of crystallinity and electrical properties, the focus shifted to investigating the potential of MDPE/GNP 1 wt% for use in vacuum environments and its final mechanical properties.

The parameters for filament extrusion were optimized, resulting in high-quality filaments with a consistent round cross-section, excellent surface finish, and precise dimensions. It was determined that the optimal chamber temperature for the extrusion is 118 °C, and the optimal screw speed is 5 rpm, corresponding to a filament extrusion speed of approximately 2 cm per minute. To mitigate warping and adhesion issues during the 3D printing, the study employed a closed chamber with a controlled temperature of 40 °C. The nozzle temperature was set at 118 °C, keeping the HDPE build plate at 80 °C. The nozzle speed was 20 mm·s^−1^, adopting a 100% infill density with a symmetric and balanced linear infill pattern ([0°/90°]_s_) for printing the tensile test specimens and concentric for the electric test samples. The slow cooling process reduced the impact of thermal history on the final 3D-printed parts. Electrical impedance data showed that 3D-printed specimens had similar electrical behavior to molded ones, indicating that the 3D printing process did not introduce defects or voids that affected the electrical properties. Tensile testing revealed that 3D-printed and molded specimens exhibited comparable tensile strength. The MDPE/xGnP 1 wt% composite showed an increase in tensile strength compared to neat MDPE. The in-space suitability of the 3D-printed polyethylene/graphene composites was tested in a simulated space environment, unveiling suitable outgassing properties. Nevertheless, further investigations are needed to fully characterize the performance of these materials under hostile space conditions.

As a future outlook, testing the 3D-printed composite samples in a relevant space environment, such as low earth orbit (LEO) or geostationary earth orbit (GEO), will further validate their suitability for space applications and advance their technology readiness level (TRL). Moreover, the optimization of the 3D printing process in microgravity conditions could be crucial to overcoming challenges related to technological advancement in space.

## Figures and Tables

**Table 1:** DSC results for neat MDPE and MDPE/xGnP composite powders. Standard deviation of data was below 2%.

Sample	T_m_ (°C)	ΔH_m_ (J/g)	T_c_ (°C)	ΔH_c_ (J/g)	χ (%)
MDPE	114.5	110.8	101.9	90.5	38.5
MDPE/xGnP 0.5 wt%	116.1	111.0	104.2	87.0	38.7
MDPE/xGnP 1 wt%	115.3	111.6	104.2	87.4	39.2
MDPE/xGnP 2 wt%	115.8	107.4	105.1	82.9	38.0
MDPE/xGnP 5 wt%	115.8	103.2	104.8	81.7	37.6

**Table 2:** Water contact angles (WCA) and surface free energies (SFE) with dispersive and polar components for 3D-printed MDPE and MDPE/xGnP 1 wt% samples.

Sample	WCA (°)	SFE (mJ/m^2^)	γ^d^ (mJ/m^2^)	γ^p^ (mJ/m^2^)
MDPE	94.7 ± 2.8	27.79	25.69	2.10
MDPE/xGnP 1 wt%	106.3 ± 3.5	31.08	31.05	0.02

**Table 3:** Optimized 3D printing parameters for neat MDPE square samples (10 mm × 10 mm × 1.5 mm) when using a glass build plate.

Process Parameter	Value
Nozzle temperature	118 °C
Build plate temperature	70 °C
Nozzle speed	20 mm·s^−1^
Infill density	100%
Infill pattern	Concentric
Layer thickness	0.1 mm

**Table 4:** Optimized 3D printing parameters for neat MDPE and MDPE/xGnP composites when using a HDPE build plate.

Process Parameter	Value
Nozzle temperature	118 °C
Build plate temperature	60 °C for MDPE, 80 °C for MDPE/xGnP 0.5 wt% and MDPE/xGnP 1 wt%
Nozzle speed	20 mm·s^−1^
Infill density	100%
Infill pattern	Lines ([0°/90°]_s_) for tensile test specimens, concentric for electric test specimens
Layer thickness	0.1 mm

**Table 5:** Results of outgassing tests with simultaneous exposure to UV irradiation (AM0 solar spectrum) of neat MDPE and composite samples. Tests were performed on 5 samples for each type, and the standard deviation of the mean value was less than 0.1. T_e_ is the equilibrium temperature measured at the back surface of the sample in the chamber during the irradiation step.

Sample	TML%	RML%	WVR%	T_e_ (°C)
MDPE	0.055	0.042	0.010	55.6
MDPE/xGnP 1 wt%	0.483	0.470	0.013	81.8

## Data Availability

Data are available from the corresponding author upon reasonable request. The data are not publicly available due to ongoing researches using a part of the data.
